# SkQ1 Suppresses the p38 MAPK Signaling Pathway Involved in Alzheimer’s Disease-Like Pathology in OXYS Rats

**DOI:** 10.3390/antiox9080676

**Published:** 2020-07-28

**Authors:** Natalia A. Muraleva, Natalia A. Stefanova, Nataliya G. Kolosova

**Affiliations:** 1 Institute of Cytology and Genetics SB RAS, 10 Lavrentieva Avenue, Novosibirsk 630090, Russia; stefanovan@mail.ru (N.A.S.); kolosova@bionet.nsc.ru (N.G.K.); 2 N. N. Vorozhtsov Institute of Organic Chemistry SB RAS, 9 Lavrentieva Avenue, Novosibirsk 630090, Russia

**Keywords:** Alzheimer’s disease, p38 MAPK, mitochondria-targeted antioxidant, SkQ1, OXYS rats

## Abstract

Alzheimer’s disease (AD) is the most common type of dementia and is currently incurable, and mitogen-activated protein kinase (MAPK) p38 is implicated in the pathogenesis of AD. p38 MAPK inhibition is considered a promising strategy against AD, but there are no safe inhibitors capable of penetrating the blood–brain barrier. Earlier, we have shown that mitochondria-targeted antioxidant plastoquinonyl-decyltriphenylphosphonium (SkQ1) at nanomolar concentrations can prevent, slow down, or partially alleviate AD-like pathology in accelerated-senescence OXYS rats. Here we confirmed that dietary supplementation with SkQ1 during active progression of AD-like pathology in OXYS rats (aged 12–18 months) suppresses AD-like pathology progression, and for the first time, we showed that its effects are associated with suppression of p38 MAPK signaling pathway (MAPKsp) activity. Transcriptome analysis, western blotting, and immunofluorescent staining revealed that SkQ1 suppresses p38 MAPKsp activity in the hippocampus at the level of expression of genes involved in the p38 MAPKsp and reduces the phosphorylation of intermediate kinases (p38 MAPK and MK2) and a downstream protein (αB-crystallin). Thus, the anti-AD effects of SkQ1 are associated with improvement in the functioning of relevant signaling pathways and intracellular processes, thus making it a promising therapeutic agent for human AD.

## 1. Introduction

Alzheimer’s disease (AD) is a progressive age-related neurodegenerative disorder in the elderly and the most prevalent cause of dementia. Today, there are no effective treatments, and therefore AD is one of the greatest healthcare challenges of our century. Existing targets of AD drug research and development include amyloid beta (Aβ), Aβ metabolism/catabolism, tau protein, inflammation, cholesterol, the cholinergic system, and other neurotransmitter systems. Nonetheless, none of them has been validated as a therapeutically effective target [1]. The hallmark of AD is defective proteostasis, namely, aggregation and accumulation of Aβ and hyperphosphorylated tau protein in neurofibrillary tangles in the brain [2,3], in combination with oxidative stress and mitochondrial dysfunction. Mitochondrial abnormalities in combination with oxidative stress are the earliest and most noticeable signs of neuronal changes in the AD brain [4]. There is strong evidence that activation of mitogen-activated protein kinase (MAPK) p38 in the human AD brain also occurs in the early stage of AD [5] and plays a substantial role in the pathogenesis of AD in the brain [6].

p38 MAPK is a class of MAPKs responsive to stress stimuli such as inflammatory cytokines and reactive oxygen species (ROS). Oxidative stress activates intracellular signaling pathways, including the p38 MAPK signaling pathway (MAPKsp) and its downstream signaling components, which, along with other factors, lead to the aggregation of Aβ and hyperphosphorylated tau protein in the brain. These highly neurotoxic aggregates are the main pathological disturbances of proteostasis in AD. They maintain p38 MAPK activation exacerbating oxidative stress [7] and provoking further deposition of protein aggregates. p38 MAPK inhibitors have been shown to be effective in both in vivo and in vitro experiments and could be used for the treatment of neurological disorders including AD [8]. Thus, inhibition of p38 MAPK is a promising strategy against AD [6,8] because p38 MAPK is a potential target for breaking the pathological Aβ toxicity cycle. Nevertheless, currently, there are no safe therapeutic agents for AD among p38 MAPK inhibitors. We hypothesized that previously proven neuroprotective effects of the mitochondria-targeted antioxidant plastoquinonyl-decyltriphenylphosphonium (SkQ1, the use of which did not have any toxic effect [9]) is associated with, inter alia, the influence on the activity of the MAPKsp.

Previously, we have shown that SkQ1 [10] at nanomolar concentrations can prevent, slow down, or partially alleviate AD-like pathology in accelerated-senescence OXYS rats [11,12]. The nontransgenic OXYS rat strain is characterized by spontaneously developing key signs of AD in humans [13]. The sequence of their development in OXYS rats is consistent with the modern understanding of the pathogenesis of the most common, i.e., sporadic (>90% of cases) form of AD: Dysfunction of mitochondria, tau protein hyperphosphorylation, an aberration of long-term post-tetanic potentiation, synaptic insufficiency, destructive changes in neurons, behavioral disorders, a decrease in cognitive functions at the early stages, aggravation of these functions during an increase in the amyloid-protein precursor level, enhanced accumulation of Aβ, and formation of Aβ plaques in the brain [12,13,14,15]. We have proved that SkQ1 can alleviate all signs of AD in OXYS rats and its effects are strongly related to the improvement of structural and functional state of mitochondria [15] as confirmed by high-throughput RNA sequencing (RNA-seq) analysis of the hippocampus of OXYS rats after dietary SkQ1 supplementation [12]. That study showed that SkQ1 affects the expression of genes associated with the structure or function of mitochondria and the key signaling pathways that are altered during the progression of AD-like pathology, especially the MAPKsp and the exclusively expressed genes related to MAPK.

Here, we studied the influence of age on p38 MAPKsp activity in the hippocampus of senescence-accelerated OXYS rats, the effect of SkQ1 supplementation during the period of active progression of signs of AD in OXYS rats (aged 12 to 18 months), and the usefulness of SkQ1 as a potential drug for AD. As one of the criteria of p38 MAPKsp activity, we evaluated changes in the p38 MAPK-dependent phosphorylation level of a small chaperone protein (αB-crystallin, i.e., CryaB) associated with senile plaques and tau-protein inclusions [16,17].

## 2. Materials and Methods

### 2.1. Ethics Statement

All experiments with the rats were authorized by Scientific Council # 9 (Institute of Cytology and Genetics, Siberian Branch, Russian Academy of Sciences [ICG SB RAS], Novosibirsk, Russia) in accordance with the Guidelines for Manipulations with Experimental Animals (Russian Academy of Sciences decree, 2 April 1980, # 12000-496).

### 2.2. Animals and Diet

To assess the age-associated alterations of the p38 MAPKsp, male OXYS rats were studied at several ages (20 days, 5 months, and 18 months) along with male Wistar rats as age-matched controls (disease-free; five per group). The rats were bred and provided by the Center for Genetic Resources of Laboratory Animals (ICG SB RAS). Rat pups were separated from mothers at age 4 weeks, were placed in cages (36 × 57 × 20 cm) at five rats per cage, and were maintained under standard conditions.

To evaluate the impact of dietary supplementation with SkQ1, 12-month-old OXYS rats were randomly distributed into two groups (eight per group). One group ate an SkQ1-supplemented diet (250 nmol per kilogram of body weight; the drug was provided on dried bread slices) every day between ages 12 and 18 months. Wistar and OXYS rats (12 months old; eight per group) that were given dried bread without the drug were used as controls. SkQ1 was made and supplied by the Institute of Mitoengineering (Moscow State University, Russia). For the RNA-seq analysis, OXYS rats after treatment with SkQ1, untreated OXYS rats, and age-matched Wistar rats (three per group) were killed by CO_2_ asphyxiation at the age of 18 months. The rest of the rats in each group were used in different experiments.

### 2.3. Tissue Preparation

The brain was excised from the rats and right away fixed in PBS containing 4% of paraformaldehyde for 48 h and then in PBS containing 30% of sucrose for 48 h at 4 °C; after that, each brain was frozen for subsequent analyses (immunostaining) in liquid nitrogen. The hippocampus of some rats (n = 8) were quickly isolated from the brain on ice, frozen in liquid nitrogen, and saved for subsequent molecular analyses. These tissue samples were kept at −70 °C before other processing.

### 2.4. Western Blotting

Hippocampi from OXYS and Wistar rats (five per group) at three ages (20 days, 5 months, and 18 months) and from 18-month-old animals after administration of SkQ1 were used for western blotting as described elsewhere [18]. Fractions of proteins (detergent-insoluble and detergent-soluble) were electrotransferred onto nitrocellulose membranes. After blocking for 1 h with a solution of 5% bovine serum albumin (BSA; Sigma–Aldrich; cat. # SLBJ8588V) in PBS containing 0.1% of Tween 20, each membrane was probed with primary antibodies overnight at 4 °C. A number of antibodies were used: An anti-MK2 (phospho T334) antibody, anti-Alpha B crystallin antibody, anti-CryaB phospho S59 antibody, anti-MK2 antibody (E341), and anti-GAPDH and anti-β-actin antibodies (Abcam, Eugene, OR, USA; cat. # ab63378, ab76467, ab5577, ab32567, ab8245, and ab1801, respectively) and anti-phospho-p38 MAPK alpha and anti-p38 MAPK alpha antibodies (cat. # 36-8500 and 33-1300, Invitrogen, Carlsbad, CA, USA). After an immunoreaction with a corresponding secondary antibody (1:5000; cat. # ab6721 and ab97046; Abcam, USA), chemiluminescence data were scanned and measured, and band intensities were quantitated using ImageJ (NIH, USA). GAPDH and β-actin were employed to set up a loading internal control.

### 2.5. Immunofluorescent Staining

Sixteen-μm-thick slices of brain tissue from Wistar and OXYS rats were put on Polysine-glass slides (Menzel-Glaser, Braunschweig, Germany) and kept in a blocking solution consisting of 1% BSA (Sigma–Aldrich, St. Louis, MO, USA) in PBS with 0.1% of Triton X-100 (PBS-T) for 1 h. Next, the slides were probed at 4 °C overnight with the primary antibody that was used for the western blot analysis and with an antibody against the amyloid β peptide (cat. # MABN254; MOAB-2; Merck Millipore, Darmstadt, Germany) diluted with the blocking solution at 1:250. After a few washings with PBS, the slides were treated at room temperature for 1 h in the dark with a secondary antibody (1:5000; cat. # ab175472, ab150170, and ab150075; Abcam, USA) diluted in a 1:300 ratio with PBS containing 1% of BSA. Then, the tissue sections were washed in PBS and coverslipped by means of a mounting medium containing 4′,6-diamidino-2-phenylindole (cat. # ab104139; Fluoro-shield with DAPI; Abcam, USA). The negative controls were subjected to the same processing except there was no primary antibody. The profiles of immunofluorescence signals were observed by fluorescence microscopy (Axioplan 2; Zeiss, Oberkochen, Germany).

### 2.6. An Enzyme-linked Immunosorbent Assay (ELISA)

The ELISA for amyloid-β1-42 (Human/Rat ELISA Kit; # 290-62601, Wako) was performed on the hippocampus of the rats in accordance with the manufacturer’s protocol. Quantitation was carried out by measuring optical density on a VICTOR multilabel plate reader (PerkinElmer Inc., Waltham, MA, USA), and the concentration was calculated in picomoles of Aβ1-42 protein per milligram of total protein of hippocampal tissue.

### 2.7. Gene Expression Analysis

The RNA-seq results on the hippocampi of 5- and 18-month-old and 20-day-old OXYS and Wistar rats and SkQ1-fed OXYS rats (n = 3 rats per group) were obtained at 18 months of age and were subjected to the processing described before [12]. At least 40 million single-end 50-bp-long reads were obtained from each specimen of hippocampus RNA via non-stranded sequencing (Illumina, San Diego, CA, USA) using Illumina GAIIx by the Genoanalitika Lab (https://www.genoanalytica.ru, Moscow), following the standard protocols of Illumina (cat. # 1004816; mRNA-Seq Sample Prep Kit). The data from sequencing were subjected to preprocessing in Cutadapt for the removal of low-quality sequences as well as adapters. The obtained sequence reads were mapped in TopHat2 to a reference genome assembly (Rnor_6.0). These data were next transformed into gene count tables using ENSEMBL and RefSeq gene annotation information. The obtained tables were studied by the analysis of differential gene expression in DESeq. Genes with a *p* value less than 0.05 were assumed to be differentially expressed.

### 2.8. Statistical Analysis

Two-way ANOVA was used to evaluate the differences between OXYS and Wistar rats across ages (age × genotype) in the Statistica 8.0 software (StatSoft, Tulsa, OK, USA). A Newman–Keuls post-hoc test was applied to significant main effects and interactions in order to estimate the differences between particular sets of means. One-way ANOVA was performed for pairwise group comparisons. Comparisons between means were carried out using one-way ANOVA. The data are presented as mean ± SEM. The differences were considered statistically significant at *p* < 0.05.

## 3. Results

### 3.1. Age-Related Changes in the Expression of Genes Associated with the p38 MAPKsp in the Hippocampus of Wistar and OXYS Rats

**Figure 1:**
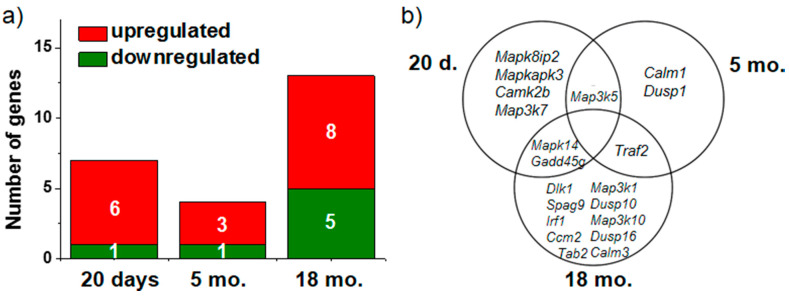
The impact of age on the number of differentially expressed genes (DEGs) involved in the p38 mitogen-activated protein kinase signaling pathway (MAPKsp) in OXYS rats (**a**). The Venn diagram shows overlapping sets of DEGs among 20-day-old and 5- and 18-month-old OXYS rats compared to age-matched Wistar rats (**b**).

We examined changes in mRNA expression of genes involved in the p38 MAPKsp in the hippocampus of Wistar and OXYS rats as a function of age. At the age of 20 days (without the manifestations of AD-like pathology), the expression of six genes (*Camk2b*, *Gadd45g*, *Map3k5*, *Mapk14*, *Mapk8ip2*, and *Mapkapk3*) was higher and *Map3k7* gene expression was lower in OXYS rats than in Wistar rats (Figure 1a). In the hippocampus of 5-month-old OXYS rats, the mRNA levels of genes *Dusp1*, *Map3k5*, and *Traf2* were higher and *Calm1* mRNA expression was lower as compared to Wistar rats. At age 18 months in OXYS rats, we identified 13 differentially expressed genes (DEGs) involved in the p38 MAPKsp. Among the genes with increased expression, we noted *Calm3*, *Ccm2*, *Dlk1*, *Gadd45g*, *Irf1*, *Map3k10*, *Mapk14*, and *Traf2*, and five genes (*Dusp10*, *Dusp16*, *Map3k1*, *Spag9*, and *Tab2*) were downregulated (Figure 1a).

### 3.2. Age-Related Alteration in p38 MAPK and Phospho-(p-)p38 MAPK Content in the Hippocampus of Wistar and OXYS Rats

**Figure 2:**
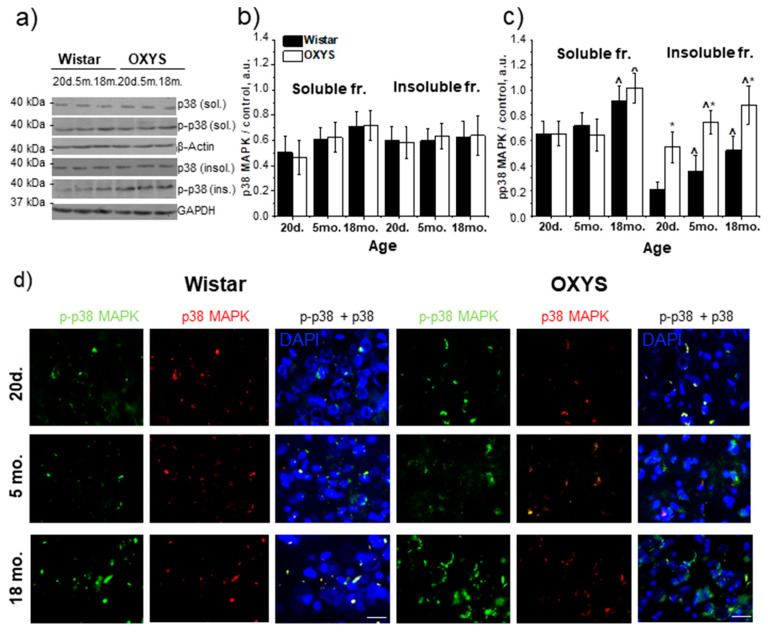
The protein content of p38 MAPK and p-p38 MAPK in the hippocampus of 20-day-old and 5- and 18-month-old Wistar and OXYS rats. (**a**) Representative western blots of total and phosphorylated p38 MAPK in the detergent-soluble and detergent-insoluble fractions from the hippocampus of Wistar and OXYS rats. Graphical presentation illustrates the relative protein content of p38 MAPK (**b**) and p-p38 MAPK (**c**) in Wistar and OXYS rats’ hippocampi at different ages after normalization of the detergent-soluble fraction data to β-actin and detergent-insoluble fraction data to GAPDH. Data are presented as mean ± SEM of five independent experiments. Immunostaining for p38 MAPK and p-p38 MAPK (**d**) in the hippocampus of 20-day-old and 5- and 18-month-old Wistar and OXYS rats. The nuclei were stained with DAPI (blue). Scale bars, 25 µm. * Statistically significant differences between the strains of the same age; ^ significant differences from the previous age within a strain (*p* < 0.05).

The western blotting revealed that the level of the p38 MAPK protein in the detergent-soluble fraction was dependent on age (F_1,24_ = 9.1, *p* < 0.01) and was the lowest at the age of 20 days in the hippocampus of both Wistar and OXYS rats (Figure 2a,b). The level of p38 MAPK in the detergent-insoluble fraction had no differences between the two rat strains and did not change with age (Figure 2a,b).

Significant changes in the magnitude of p38 MAPK phosphorylation were identified in the hippocampus of both Wistar and OXYS rats (Figure 2a–c). In the detergent-soluble fraction, this magnitude depended only on age (F_1,24_ = 24.1, *p* < 0.01), and in the hippocampus of 18-month-old rats of both strains was higher than the level at the age of 20 days and 5 months. The level of the p-p38 MAPK in the detergent-insoluble fraction increased with age (F_2,24_ = 19.7, *p* < 0.01) in Wistar and OXYS rats and was affected by the strain (i.e., genotype; F_1,24_ = 74.4, *p* < 0.01). The amount of p-p38 MAPK was significantly higher in the hippocampus of OXYS rats than Wistar rats in all age groups (*p* < 0.01, *p* < 0.01, and *p* < 0.03, respectively). The western blotting data were supported by immunostaining results (Figure 2d).

### 3.3. Age-Related Alterations of CryaB and p-Ser59-CryaB Protein Content in the Rat Hippocampus

**Figure 3:**
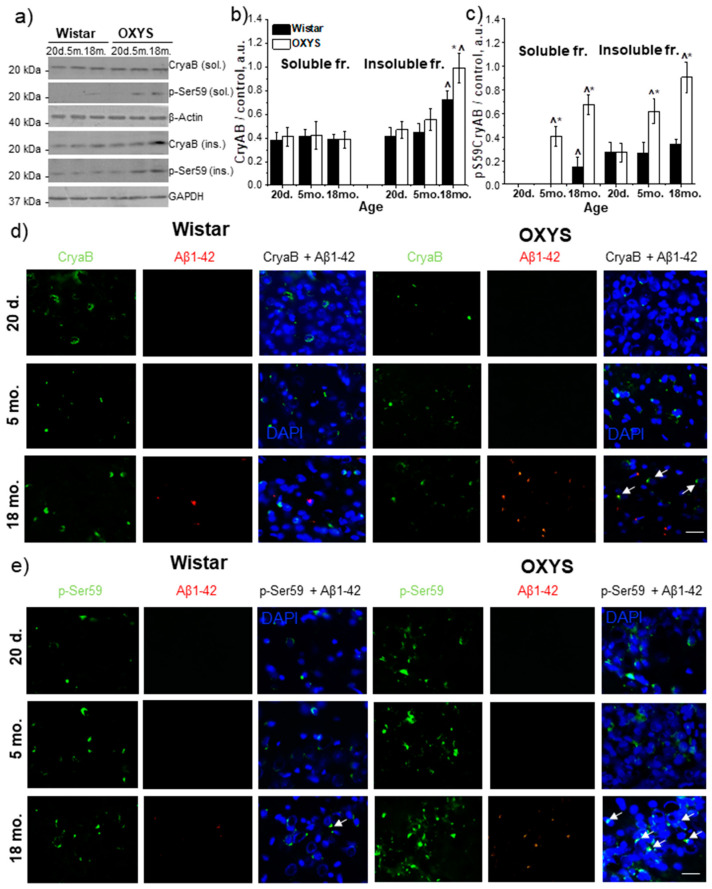
The amounts of proteins CryaB and p-Ser59-CryaB in the hippocampus of 20-day-old and 5- and 18-month-old Wistar and OXYS rats. (**a**) Representative western blots of total and phosphorylated CryaB in the detergent-soluble and detergent-insoluble fractions from the hippocampus of Wistar and OXYS rats. Graphical presentation shows the relative protein content of CryaB (**b**) and p-Ser59-CryaB (**c**) in Wistar and OXYS rats’ hippocampi at different ages after normalization of detergent-soluble fraction data to β-actin and detergent-insoluble fraction data to GAPDH. Data are presented as mean ± SEM of five independent experiments. Immunostaining of CryaB (**d**) and p-Ser59-CryaB (**e**) in the hippocampus of 20-day-old and 5- and 18-month-old Wistar and OXYS rats. The nuclei were stained with DAPI (blue). Scale bars, 25 µm. * Statistically significant differences between the strains of the same age; ^ significant differences from the previous age within a strain (*p* < 0.05).

As a target protein phosphorylated by components of the p38 MAPK pathway, we examined the expression of molecular chaperone CryaB and the level of its phosphorylation at the Ser59 position. The amounts of CryaB and p-Ser59-CryaB proteins were determined in the detergent-soluble and detergent-insoluble fractions from the hippocampus of 20-day-old and 5- and 18-month-old Wistar and OXYS rats (Figure 3a–c). We did not detect age-related changes and interstrain differences in the level of the CryaB protein in detergent-soluble fractions. At the same time, in the detergent-insoluble fraction, CryaB content was dependent on genotype and age (F_1,24_ = 20.8; *p* < 0.01 and F_2,24_ = 66.9; *p* < 0.01, respectively). The factors of age and genotype interacted (F_2,24_ = 4.2; *p* < 0.03). CryaB content increased significantly by the age of 18 months in rats of both strains and was higher in OXYS rats (*p* < 0.01).

The p-Ser59-CryaB protein was found in both protein fractions in Wistar and OXYS rats (Figure 3a,c). According to ANOVA, p-Ser59-CryaB content in the detergent-soluble fraction was affected by genotype (F_1,24_ = 210.8, *p* < 0.01) and age (F_2,24_ = 122.6, *p* < 0.01), and there was an interaction effect (F_2,24_ = 55.4, *p* < 0.01). We did not detect the p-Ser59-CryaB protein in the detergent-soluble fraction from the hippocampi of 20-day-old Wistar and OXYS rats and of 5-month-old Wistar rats (Figure 3a,c). In rats of both strains, the p-Ser59-CryaB protein content increased with age, and at the age of 18 months, it was the highest. In addition, its amounts were higher in 5- and 18-month-old OXYS rats (*p* < 0.01 and *p* < 0.05, respectively). The amount of detergent-insoluble p-Ser59-CryaB at the age of 20 days did not differ between OXYS and Wistar rats (*p* > 0.05) but increased with age in both rat strains (Figure 3a,c). Its level was higher in 5- and 18-month-old OXYS rats (*p* < 0.01 and *p* < 0.05, respectively) than in respective Wistar rats. The western blotting data were supported by immunostaining results (Figure 3d,e). A significant increase in the colocalization of p-Ser59-CryaB with Aβ_1–42_ was revealed in 18-month-old OXYS rats.

### 3.4. SkQ1 Prevents Accumulation of Aβ in the Hippocampus of OXYS Rats

The ELISA confirmed the significantly elevated level of Aβ_1–42_ in the hippocampi of 18-month-old untreated OXYS rats (147.0 ± 4.47 pg/(mg of protein)) compared to the control Wistar rats (95.8 ± 9.32 pg/(mg of protein)). SkQ1 significantly lowered the protein level of Aβ_1–42_ in OXYS rats (112.9 ± 7.14) in comparison with untreated Wistar and OXYS rats (*p* < 0.001 and *p* < 0.006, respectively).

### 3.5. SkQ1 Restores the Expression of Genes Associated with the p38 MAPKsp in the Hippocampus of OXYS Rats

**Figure 4:**
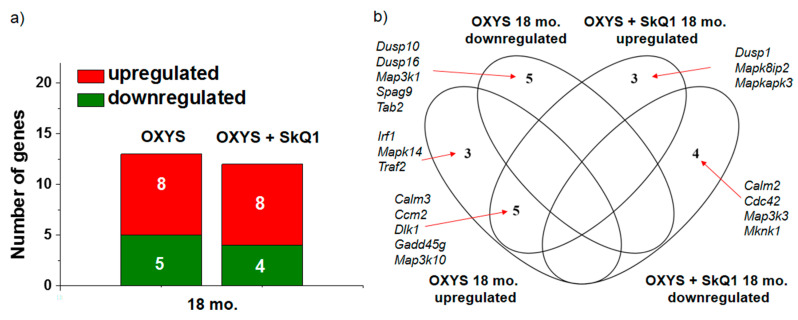
The influence of supplementation with SkQ1 in OXYS rats on the number of DEGs among those known to be involved in the p38 MAPKsp (**a**). The Venn diagram depicts overlapping sets of DEGs in control and SkQ1-treated 18-month-old OXYS rats compared of untreated Wistar rats (**b**).

Supplementation with SkQ1 changed the DEGs and their total number, among those that are known to be involved in the p38 MAPKsp (Figure 4a). Supplementation with SkQ1 normalized the expression of genes *Irf1*, *Mapk14*, and *Traf2* (whose mRNA levels were high in untreated OXYS rats; Figure 4b) and increased the expression of genes *Dusp1*, *Mapk8ip2*, and *Mapkapk3*. While the rats were taking SkQ1, the expression of all five downregulated genes (*Dusp10*, *Dusp16*, *Map3k1*, *Spag9*, and *Tab2*) increased to the level of control animals. The mRNA levels of four genes (*Calm2*, *Cdc42*, *Map3k3*, and *Mknk1*) significantly decreased in SkQ1-treated OXYS rats relative to untreated OXYS rats.

### 3.6. SkQ1 Prevents Accumulation of Proteins p38 MAPK and p-p38 MAPK in the Hippocampus of OXYS Rats

**Figure 5:**
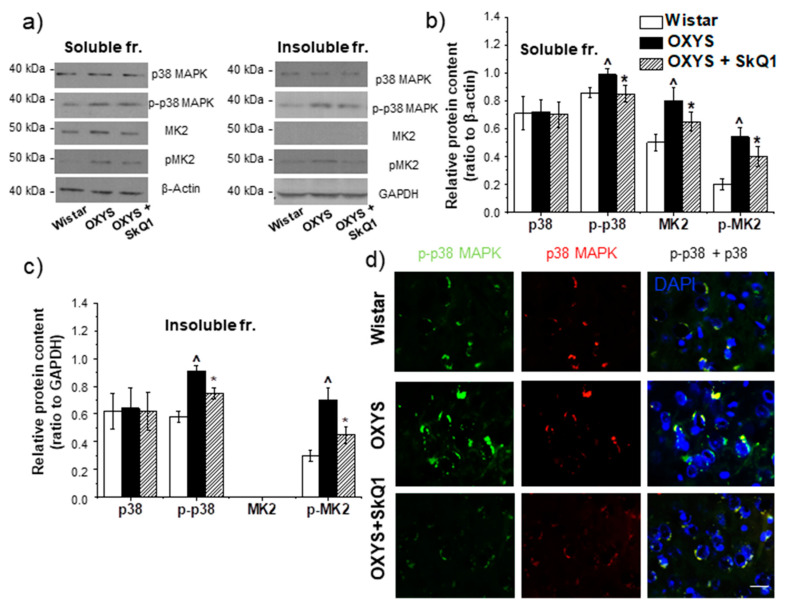
The impact of SkQ1 supplementation in OXYS rats from age 12 to 18 months on the amounts of proteins p38 MAPK, p-p38 MAPK, MK2, and p-MK2 in the hippocampus. Representative western blots of total and phosphorylated p38 MAPK and MK2 in the detergent-soluble and detergent-insoluble fractions in the hippocampus of untreated Wistar rats and OXYS, and OXYS rats with SkQ1 (**a**). Graphical presentation depicts relative protein content of p38 MAPK, p-p38 MAPK, MK2, and p-MK2 in the hippocampi of untreated Wistar and OXYS rats and in OXYS rats taking SkQ1 after normalization to β-actin for the detergent-soluble protein fraction (**b**) and normalization to GAPDH for the detergent-insoluble fraction (**c**). Data are presented as mean ± SEM of five independent experiments. Immunostaining of p38 MAPK and p-p38 MAPK (**d**) in the hippocampus of untreated Wistar and OXYS rats and OXYS rats taking SkQ1. The nuclei were stained with DAPI (blue). Scale bars, 25 µm. ^ Statistically significant differences between the strains of the same age; * the effect of supplementation with SkQ1 (*p* < 0.05).

Supplementation with SkQ1 had no effect on the p38 MAPK content in the hippocampus of OXYS rats but significantly changed the level of phosphorylation of p38 MAPK in both protein fractions (Figure 5a–c). In the detergent-soluble fraction from OXYS rats taking SkQ1, one-way ANOVA revealed a decrease in p-p38 MAPK content (*p* < 0.01) to the level of control Wistar rats. In the detergent-insoluble fraction, we noted a similar decrease in p-p38 MAPK content, but here, the level of phosphorylation in OXYS rats taking SkQ1 did not reach the level of Wistar rats (Figure 5a–c). The western blotting data were supported by immunostaining findings (Figure 5d).

### 3.7. The Effect of SkQ1 on MK2 and p-MK2 Protein Contents in the Hippocampus of OXYS Rats with AD-Like Pathology

Western blot analysis revealed that the MK2 protein content in the detergent-soluble fraction from OXYS rats’ hippocampi was higher than that in Wistar rats (*p* < 0.02; Figure 5a–b), and dietary supplementation with SkQ1 significantly reduced this level in OXYS rats (*p* < 0.01; Figure 5a–b). In the detergent-insoluble fractions, the MK2 protein was absent in both rat strains (Figure 5a,c). The phosphorylated form of MK2 was detected in both protein fractions from the hippocampus of 18-month-old OXYS and Wistar rats. Its protein amount was higher in the hippocampus of OXYS rats than the hippocampus of Wistar rats in the detergent-soluble (*p* < 0.02; Figure 5a,b) and detergent-insoluble fractions (*p* < 0.02; Figure 5a,c). The dietary supplementation with SkQ1 significantly reduced this parameter in OXYS rats in both protein fractions (*p* < 0.01 and *p* < 0.01, respectively; Figure 5a–c).

### 3.8. The Effect of SkQ1 on the CryaB and p-Ser59-CryaB Protein Content in the Hippocampus of OXYS Rats

**Figure 6:**
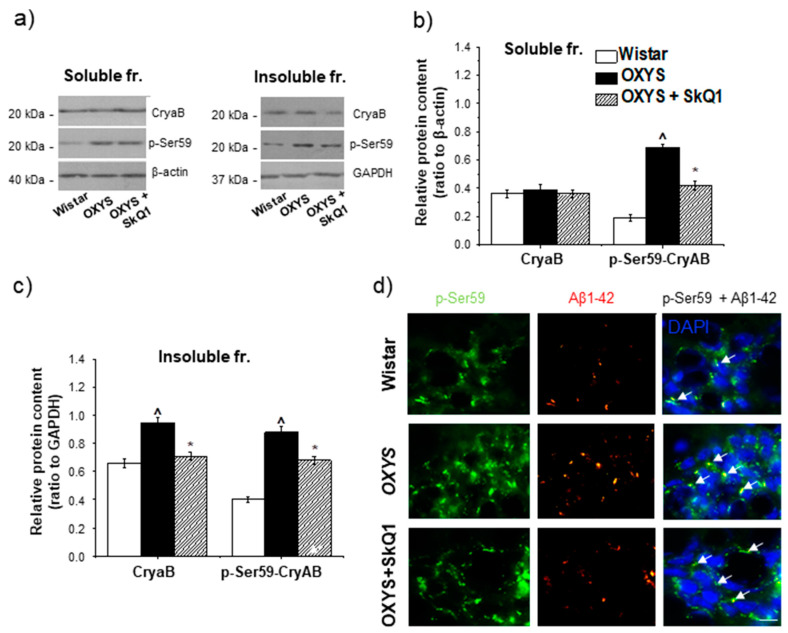
The effect of SkQ1 supplementation in OXYS rats from age 12 to 18 months on the protein levels of CryaB and p-Ser59-CryaB in the hippocampus. Representative western blots of total and phosphorylated CryaB in the detergent-soluble and detergent-insoluble fractions from the hippocampus of untreated Wistar and OXYS rats and OXYS rats treated with SkQ1 (**a**). Graphical presentation illustrates the relative levels of proteins CryaB and p-Ser59-CryaB in the hippocampus of untreated Wistar and OXYS rats and OXYS rats taking SkQ1 after normalization to β-actin for the detergent-soluble protein fraction (**b**) and to GAPDH for the detergent-insoluble fraction (**c**). Data are presented as mean ± SEM of five independent experiments. Immunostaining for p-Ser59-CryaB (**d**) in the hippocampus of untreated Wistar and OXYS rats and OXYS rats taking SkQ1. The nuclei were stained with DAPI (blue). Scale bars, 25 µm. ^ Statistically significant differences between the strains of the same age; * the effect of supplementation with SkQ1 (*p* < 0.05).

The effect of SkQ1 supplementation on the CryaB content was detectable only in the detergent-insoluble fraction. This parameter was lower in SkQ1-treated OXYS rats than in untreated OXYS rats (*p* < 0.01; Figure 6a–c) but did not reach the lower level of control Wistar rats (*p* < 0.03; Figure 6a–c). Statistical analysis indicated that SkQ1 reduced the protein content of p-Ser59-CryaB in the detergent-soluble and detergent-insoluble protein fractions of the hippocampus from OXYS rats (*p* < 0.01 and *p* < 0.01, respectively; Figure 6a–c), but this parameter remained significantly higher than that in untreated Wistar rats (*p* < 0.01 and *p* < 0.01, respectively). The western blotting data were supported by immunostaining results (Figure 6d).

## 4. Discussion

Earlier, we have reported that treatment with SkQ1 starting from the predementia phase of AD (consistent with the definition of progressive, amnestic mild cognitive impairment in humans) prevents the neuronal loss and synaptic damage, enhances neurotrophic supply, and decreases Aβ_1–42_ peptide levels and tau hyperphosphorylation in the hippocampus of OXYS rats, as well as improving the structural and functional state of mitochondria, thus improving the learning ability and memory [12,13,14,15]. In the present study, by quantifying Aβ in the hippocampus, we confirmed that dietary supplementation with SkQ1 in this age period (from 12 to 18 months) suppresses the progression of AD-like pathology in OXYS rats (Table 1). For the first time, we showed that these effects are associated with the suppression of the p38 MAPKsp activity. In addition, we provide compelling evidence that the progression of AD-like pathology in senescence-accelerated OXYS rats proceeds simultaneously with alterations in p38 MAPKsp activation and an increase in p38 MAPK-dependent CryaB phosphorylation in the hippocampus.

According to our RNA-seq analysis, activation of the p38 MAPKsp in the hippocampus of OXYS rats occurs at an early age, before clinical manifestations of AD-like pathology. Among upregulated DEGs, we noted the *Mapk14* gene (mitogen-activated protein kinase 14), which encodes the p38 MAPK protein and is a key gene of the p38 MAPKsp. *Gadd45g* (growth arrest and DNA damage-inducible gamma) and *Map3k5* (mitogen-activated protein kinase kinase kinase 5) serve as activators of the p38 MAPKsp by mediating phosphorylation and subsequent activation of the upstream kinases thereby confirming the pathway activation. The *Mapk8ip2* gene encodes a scaffold protein that is thought to participate in the regulation of the c-Jun amino-terminal kinase signaling cascade. MAPKAPK3 positively regulates autophagy [19]. All the respective genes are related to positive regulation of the p38 MAPKsp, and an increase in their expression indicates activation of this signaling pathway. Among the genes upregulated in OXYS rats at the age of 5 months, *Map3k5* and *Dusp1* have a different effect on p38 MAPKsp activity. The protein encoded by *Dusp1* (dual specificity phosphatase 1) participates in the negative control of MAPK signaling activity [20]. The increase in *Dusp1* expression may be regarded as a compensatory response to changes of p38 MAPK signaling in OXYS rats at the stage of manifestation of AD-like pathology. The progression of AD-like pathology in OXYS rats was linked with an increase in the number of DEGs among those known to be involved in the p38 MAPKsp. Among them, genes *Gadd45g*, *Map3k10*, and *Mapk14* are activators of signal transduction. Moreover, we also documented reduced expression of genes *Dusp10* (dual specificity phosphatase 10) and *Dusp16* (dual specificity phosphatase 16) that inactivate signal transduction [21]. The expression of the *MAPK14* gene encoding the p38α MAPK protein turned out to be increased in the hippocampus of OXYS rats before the manifestation of AD-like pathology signs and during their active progression. Nevertheless, we did not notice significant changes in the p38 MAPK protein content with age. It can be assumed that the stimulation of protein synthesis was accompanied by an increase in the amount of p38 MAPK in the hippocampus and by activation of its phosphorylation. Indeed, the level of p38 MAPK phosphorylation was high at the preclinical stage of the disease, and this level was even higher in OXYS rats during the development of AD-like pathology.

The p38 MAPK protein is one of the most important kinases of the cascade that involves signal transduction from the cell membrane to the nucleus [22]. One feature of this cascade is that it consists of three kinases (MAP3K, MAPKK, and MAPK) that are activated through sequential phosphorylation and ultimately cause the phosphorylation of target regulatory proteins. This event serves as an indicator of both signal transmission efficiency and signaling pathway activity in general. In our study, we evaluated p38 MAPK-dependent phosphorylation of CryaB (at serine position 59), which is a small chaperone protein associated with senile plaques and tau protein inclusions [16,17]. In the hippocampi of OXYS rats, the amount of p-Ser59-CryaB was high during the period of manifestation of AD signs (age of 5 months) and further increased in the period of active progression of AD-like pathology (age of 18 months). Our results are consistent with the finding that in postmortem brain samples from patients with AD, p-Ser59-CryaB content is significantly increased [23]. Recently, we showed that the level of p-Ser59-CryaB increases in the prefrontal cortex of OXYS rats in the corresponding age period [18]. Nevertheless, we did not find p-Ser59-CryaB in the detergent-soluble fraction from the prefrontal cortex, while it is present in the hippocampus. On the one hand, this result may indicate a difference in the severity of neurodegenerative changes between the prefrontal cortex and hippocampus. On the other hand, the p-Ser59-CryaB content may be significantly lower in the prefrontal cortex than in hippocampus and cannot be detected by the semiquantitative method of western blotting.

The increase of p-Ser59-CryaB content in the insoluble fraction reflects a shift in the protective strategies of the cell for preventing aggregation and for isolation of dangerous types of proteins. It has been suggested that if denatured proteins cannot be refolded or degraded, then the resulting protein molecules are more toxic to the cell than insoluble aggregates [24]. Multiple reports indicate the binding and interaction of CryaB with Aβ [16,25], which may be enhanced by CryaB phosphorylation. In the present study, we demonstrated that p-Ser59-CryaB more often than CryaB is colocalized with toxic Aβ_1–42_ in the hippocampus of 18-month-old OXYS rats. These results are consistent with our previous finding of p-Ser59-CryaB colocalization with Aβ in the prefrontal cortex of OXYS rats at the later stages AD-like pathology [18]. Thus, we provided compelling evidence that the progression of AD-like pathology in senescence-accelerated OXYS rats takes place during activation of the p38 MAPKsp.

Lately, decreasing the accumulation of Aβ aggregates is considered a promising strategy for the treatment of AD. Here we confirmed that prolonged dietary supplementation with SkQ1 (from age 12 to 18 months) delays the accumulation of pathological Aβ in the OXYS hippocampus.

According to the RNA-seq analysis, the supplementation with SkQ1 did not exert a significant effect on the number of DEGs among those involved in the signaling pathway but significantly changed the set of these genes, and this change indicates a decrease in p38 MAPKsp activity. Against the background of SkQ1 administration, mRNA expression of *Mapk14* (an important gene of the p38 MAPKsp) was normalized, whereas in untreated OXYS rats, it proved to be upregulated. The attenuation of pathway activity was confirmed by increased expression of negative regulators (including *Dusp1)* of MAPKsp activity. Taylor and colleagues have demonstrated that this change can inhibit toxic MAPK-mediated effects while preserving the protective mechanisms of MAPK signaling [20]. In addition, the expression of two more genes (*Dusp10* and *Dusp16*) of this family—which performs negative regulation of the p38 MAPKsp [21]—was found to normalize in the OXYS rats taking SkQ1.

Data from numerous studies support the role of p38 MAPKsp activation in neuronal apoptosis in AD [8]. In this regard, the impact of prolonged administration of SkQ1 on the expression of *Map3k1* mRNA (mitogen-activated protein kinase kinase kinase 1) is interesting. This gene is an activator of MAPK pathways with an antiapoptotic effect. Supplementation with SkQ1 increased its expression to the level of control animals. Meanwhile, in the group of OXYS rats receiving SkQ1, there was upregulation of the *Mapkapk3* (MAPK activated protein kinase 3) gene, which positively regulates autophagy. Supplementation with SkQ1 did not affect several DEGs that are p38 MAPKsp activators. Among them, *Gadd45g* participates in the development of the nervous system through mechanisms of cell cycle regulation and apoptosis [26], and *MAP3K10* takes part in the regulation adult neurogenesis. Their expression remained elevated in the group of OXYS rats treated with SkQ1.

The differential expression of the *Mapk14* gene was detected in OXYS rats at the ages of 20 days and 18 months. In addition, the supplementation with SkQ1 reduced the expression of this gene in OXYS rats; therefore, the p38 MAPK protein was selected as the key indicator in our study. Earlier, we have shown that in the cerebral cortex of OXYS rats, the development of signs of Alzheimer’s disease proceeds during an increase in p38 MAPK-dependent CryaB phosphorylation (p-s59-CryaB) without increasing expression of the gene encoding CryaB. In our experiment, we studied the effect of a diet with SkQ1 supplementation on signaling pathway activity by measuring the expression of genes involved in the p38 MAPKsp and phosphorylation of intermediate kinases (MK2) and a downstream target protein (CryaB).

The influence of prolonged supplementation with SkQ1 on the p38 MAPKsp was confirmed here at the molecular level. Even though total p38 MAPK content was not affected by the drug, a significant decrease in the level of p38 MAPK phosphorylation was detected in the group of OXYS rats taking SkQ1. Decreased phosphorylation of p38 MAPK is direct evidence of decreased MAPKsp activity [8]. Within the cell, the p38 MAPK protein is present in soluble form in the cytoplasm and/or nucleus or is associated with membrane components. Currently, there is no clear evidence that p38 MAPK activation through its phosphorylation is associated with a change in p38 MAPK protein localization. In our experiment, the effect of SkQ1 on the level of p38 MAPK phosphorylation was similar between the soluble and insoluble fraction, and this finding may indicate high bioavailability of the drug. In addition, p38 MAPK suppression diminished the phosphorylation of its downstream target CryaB. It is known that p38 MAPK regulates CryaB phosphorylation by stimulating MK2 (MAPK activated protein kinase 2). It is one of the important substrates of p38 MAPK and is independently regulated by the p38 MAPKsp, followed by downstream phosphorylation events and consequent regulation of cellular functions of these proteins [27]. MK2 and p-MK2 amounts were lower in the hippocampus of the OXYS rats taking SkQ1 than in untreated OXYS rats (Figure 5).

Thus, the inhibitory effect of SkQ1 supplementation was demonstrated by measuring the expression of genes involved in the p38 MAPKsp and phosphorylation of intermediate kinases and a downstream target protein. On the basis of our findings, we can hypothesize that the neuroprotective mechanism of action of this mitochondria-targeted antioxidant on AD-like pathology of OXYS rats is mediated by direct suppression of MAPKsp activity. The accumulation of neurotoxic protein aggregates is a pathological sign of AD that causes neuronal damage through oxidative stress, caspase activation, and mitochondrial dysfunction. Considering the significant association of p38 MAPKsp activation with Aβ aggregation, inhibitors of this signaling pathway should influence the dynamics of toxic-aggregate accumulation. Indeed, the prolonged supplementation with SkQ1 in our experiment slowed down the accumulation of pathological Aβ in the OXYS rat hippocampus. Similar results about the treatment and prevention of AD-like pathology by SkQ1 have been obtained in several other studies on SkQ1’s effects on the dynamics of neurodegenerative changes in OXYS rats [11,13,15]. In addition, these studies have revealed that this drug reduced the overall level of the hyperphosphorylated tau protein, owing to a decrease in its phosphorylation on MAPK-dependent sites [13,15].

Nevertheless, direct suppression of the p38 MAPKsp, which leads to lower accumulation of pathological Aβ and hyperphosphorylated tau, does not explain all the effects of SkQ1 on neurodegeneration in OXYS rats. Oxidative stress can induce or mediate the activation of MAPK pathways [28]. One would expect that the effects of SkQ1 as an antioxidant are mediated by the suppression of ROS production. On the other hand, we have previously shown that the development of pathological signs in rats is accompanied by increasing mitochondrial dysfunction but not an increase in ROS production [29]. The therapeutic effects of supplementation with SkQ1 on the AD-like pathology in OXYS rats are associated with improvement of the mitochondrial apparatus and better neurotrophic supply [11,15]. Accumulating in mitochondria of brain neurons [15], SkQ1 normalizes the expression of genes associated with mitochondria [12]. Ultimately, the reversal of structural and functional damage to mitochondria should decrease the accumulation of neurotoxic aggregates and promote the suppression of p38 MAPKsp activity. 

In addition, dietary supplementation with SkQ1 in OXYS rats suppresses the development of the retinopathy similar to age-related macular degeneration in humans: A late-onset neurodegenerative retinal disease that shares several clinical and pathological features with AD [30,31]. One of them is believed to be aberrant proteostasis with accumulation of aggregated proteins. It is noteworthy that the therapeutic effect of SkQ1 is associated with a slowdown of Aβ accumulation and significantly decreased activity of mTOR signaling in the retina of OXYS rats [32].

Thus, in our study, we showed that the mitochondrial antioxidant SkQ1 reduces the accumulation of Aβ in the hippocampus of senescence-accelerated OXYS rats during AD-like pathology by suppressing p38 MAPKsp activity. Besides, we confirmed the previously reported finding that the anti-AD effects of mitochondria-targeted antioxidant SkQ1 are not directly related to its antioxidant activity but rather are associated with an improvement in the functioning of many signaling pathways and intracellular processes.

## Figures and Tables

**Table 1:** The influence of supplementation with SkQ1 on Aβ_1–42_ concentration in the hippocampus of OXYS rats.

	Wistar	OXYS	OXYS + SkQ1
Aβ_1–42_, pg/mg of protein	95.8 ± 9.32	147.0 ± 4.47 ^	112.9 ± 7.14 *

^ Statistically significant differences between the strains of the same age; * the effect of supplementation with SkQ1 (*p* < 0.05).
